# *Wecomedon* Jarrett & Bousfield, 1982, a replacement name for the amphipod genus *Psammonyx* Bousfield, 1973 (Crustacea, Amphipoda, Tryphosidae), preoccupied by *Psammonyx* Döderlein, 1892 (Foraminifera, Astrorhizida, Ammovolummidae)

**DOI:** 10.3897/zookeys.750.24529

**Published:** 2018-04-16

**Authors:** Tammy Horton

**Affiliations:** 1 National Oceanography Centre, Southampton, European Way, Southampton SO14 3ZH, UK

**Keywords:** *Psammonyx*, *Wecomedon*, Amphipoda, Foraminifera, homonym

## Abstract

*Wecomedon* Jarrett & Bousfield, 1982 is reintroduced as a replacement name for *Psammonyx* Bousfield, 1973 (Crustacea: Amphipoda: Tryphosidae) which is a junior homonym of *Psammonyx* Döderlein, 1892 (Foraminifera: Astrorhizida: Ammovolummidae).

## Introduction

*Psammonyx* Bousfield, 1973 was established as a new genus of lysianassid amphipod (now in the family Tryphosidae) with its type species, *Anonyx nobilis* Stimpson, 1853. It was recently discovered to be a junior homonym of *Psammonyx* Döderlein, 1892, a genus of ammovolummid foraminiferan (Foraminifera: Astrorhizida: Ammovolummidae). *Psammonyx* Döderlein, 1892 is a valid name in current use and contains the type species *Psammonyx vulcanicus* Döderlein, 1892 along with two further fossil species; *Psammonyx campbelli* Browne & Schott, 1963 and *Psammonyx maxwelli* Ireland, 1939 (Hayward et al. 2018).

## Systematics

### Wecomedon

Taxon classificationAnimaliaAstrorhizidaAmmovolummidae

Jarrett & Bousfield, 1982, re-established

Psammonyx Bousfield, 1973: 144 (Crustacea: Amphipoda: Tryphosidae); type species: Anonyx nobilis Stimpson, 1853, original designation by Bousfield 1973; preoccupied.
Wecomedon Jarrett & Bousfield, 1982: 113 (Crustacea: Amphipoda: Tryphosidae); type species: Hippomedon wecomus J.L. Barnard, 1971, original designation by Jarrett and Bousfield 1982.
non Psammonyx Döderlein, 1892: 146 (Foraminifera: Astrorhizida: Ammovolummidae); type species: Psammonyx vulcanicus Döderlein, 1892, original designation by Döderlein 1892.
Psammonyx – Budnikova, 2005: 179 (Wecomedon Jarrett & Bousfield, 1982 reduced to the rank of junior synonym of Psammonyx Bousfield, 1973).

#### Type species.

*Hippomedon wecomus* J.L. Barnard, 1971, original designation by Jarrett and Bousfield (1982).

#### Remarks.

*Psammonyx* Bousfield, 1973 currently contains eleven species (Horton et al. 2018). The genus was established by Bousfield in 1973, for the single species *Psammonyx nobilis* (Stimpson, 1853), designated as the type of the genus (as *Anonyx nobilis* Stimpson, 1853). A second species (*P. terranovae* Steele, 1979) was added by Steele in 1979, and in 1982, Jarrett and Bousfield described a third species (*Psammonyx longimerus* Jarrett & Bousfield, 1982) and transferred the fourth species *Psammonyx kurilicus* (Gurjanova, 1962) from the genus *Hippomedon*.

In 2005, Budnikova added two further species (*Psammonyx kudrjaschovi* Budnikova, 2005 and *Psammonyx tzvetkovae* Budnikova, 2005) and placed the genus *Wecomedon* Jarrett & Bousfield, 1982 in synonymy with *Psammonyx*, transferring the five species it contained to *Psammonyx* (*P. boreopacificus* (Gurjanova, 1962); *P. minusculus* (Gurjanova, 1938) *P. similis* (Jarrett & Bousfield, 1982); *P. wecomus* (J.L. Barnard, 1971); and *P. wirketis* (Gurjanova, 1962)).

According to ICZN article 60.2 the oldest available and potentially valid synonym of the rejected junior homonym becomes the valid name of the taxon (see ICZN Article 23.3.5), and takes its own authorship and date. Therefore, the replacement name for *Psammonyx* Bousfield, 1973 is *Wecomedon* Jarrett & Bousfield, 1982, with *Hippomedon wecomus* J.L. Barnard, 1971 as the type species of the genus.

***Wecomedon* is now comprised of the following eleven species**:

*Wecomedon boreopacificus* (Gurjanova, 1962), **comb. n.** Original name *Hippomedon boreopacificus*;

*Wecomedon kudrjaschovi* (Budnikova, 2005), **comb. n.** Original name *Psammonyx kudrjaschovi*;

*Wecomedon kurilicus* (Gurjanova, 1962), **comb. n.** Original name *Hippomedon kurilicus*;

*Wecomedon longimerus* (Jarrett & Bousfield, 1982), **comb. n.** Original name *Psammonyx longimerus*;

*Wecomedon minusculus* (Gurjanova, 1938), **comb. n.** Original name *Paratryphosites minusculus*;

*Wecomedon nobilis* (Stimpson, 1853), **comb. n.** Original name *Anonyx nobilis*;

*Wecomedon similis* Jarrett & Bousfield, 1982, **comb. n.** Original name *Wecomedon similis*;

*Wecomedon terranovae* (Steele, 1979), **comb. n.** Original name *Psammonyx terranovae*;

*Wecomedon tzvetkovae* (Budnikova, 2005), **comb. n.** Original name *Psammonyx tzvetkovae*;

*Wecomedon wecomus* (J.L. Barnard, 1971), **comb. n.** Original name *Hippomedon wecomus*;

*Wecomedon wirketis* (Gurjanova, 1962), **comb. n.** Original name *Hippomedon wirketis.*

## Supplementary Material

XML Treatment for Wecomedon
